# Influence of surgical factors on patient satisfaction after bi‐cruciate stabilized total knee arthroplasty: retrospective examination using multiple regression analysis

**DOI:** 10.1186/s12891-021-04098-8

**Published:** 2021-02-23

**Authors:** Hiroshi Inui, Shuji Taketomi, Ryota Yamagami, Kenichi Kono, Kohei Kawaguchi, Kosuke Uehara, Sakae Tanaka

**Affiliations:** Department of Orthopaedic Surgery, Faculty of Medicine, The University of Tokyo, 7-3-1 Hongo, Bunkyo-ku, 113-0033 Tokyo, Japan

**Keywords:** Bi‐cruciate stabilization, Medial joint stability, Multivariate regression analysis, Patient satisfaction, Total knee arthroplasty

## Abstract

**Background:**

One of the causes of dissatisfaction following total knee arthroplasty (TKA) is abnormal knee kinematics. A newly designed bi-cruciate stabilized (BCS) TKA system has been developed to produce close-to-normal kinematics because of its anatomic tibiofemoral articular geometry and cam-post mechanism. Although BCS TKA is expected to improve patient satisfaction, no reports have described the appropriate technique or soft tissue handling required to achieve excellent satisfaction with BCS TKA. This study is to identify intraoperative surgical predictors of patient satisfaction after BCS TKA.

**Methods:**

We studied 104 knees with primary varus knee osteoarthritis that underwent BCS TKA with a navigation system retrospectively. Surgical parameters including coronal, sagittal and axial alignment and joint laxity in each compartment that affected patient satisfaction was evaluated. Satisfaction score was evaluated with use of the 2011 Knee Society Scoring system. The multivariate regression analysis included age, gender, body mass index and intraoperative parameters that correlated with satisfaction scores in the univariate analysis (*P* < 0.05). The current study focused on the patient satisfaction score at 1 year postoperatively and didn’t evaluate the long term clinical results nor survivorship.

**Results:**

The postoperative satisfaction score was 28.6 ± 8.1. Multivariate analysis showed that medial joint laxity at 30° flexion (*P* = 0.003), tibial excessive external rotation alignment (*P* = 0,009) and tibial varus alignment (*P* = 0.029) were predictors of poor satisfaction score.

**Conclusions:**

When performing BCS TKA, surgeons should pay attention to maintaining proper stability of the medial compartment at mid flexion range and should avoid tibial varus and excessive external rotational alignment.

## Background

Total knee arthroplasty (TKA) is the definitive and best technique for addressing late-stage osteoarthritis (OA) in the knee. Prosthetic survival has been extended gradually, with fewer postoperative complications, with advances in prosthesis technology. Nevertheless, about 20 % of TKA patients are reportedly dissatisfied with their surgically restored knees [1, 2].

One of the causes for dissatisfaction with TKA is reported to be abnormal knee kinematics, such as a lack of tibial rotation during flexion and paradoxical motion, indicating that there is anterior sliding of the femoral component on the tibia as the knee goes into flexion, largely due to the posterior position of the femur relative to the tibia during full extension [3].

Recently, a newly designed bi-cruciate stabilized (BCS) total knee system (Journey I BCS; Smith & Nephew, Memphis, TN, USA) has been developed to create close-to-normal kinematics. This prosthesis substitutes for the lack of cruciate ligaments and menisci by restoring anatomic tibiofemoral articular geometry and placing a cam-post mechanism [4, 5]. Modifying Journey I BCS into the Journey II BCS system to avoid complications (e.g., iliotibial band [ITB] friction syndrome, dislocation) has resulted in close-to-normal kinematics and has led to good clinical and functional short-term results [6–8].

To our knowledge, there has been no previous report which describes the appropriate surgical technique required to achieve excellent patient satisfaction after BCS TKA. Surgeons should be aware of BCS TKA techniques to increase patient satisfaction.

Therefore, this study aimed to identify intraoperative predictors of patient satisfaction following BCS TKA.

## Methods

 The institutional review board approved this study. All patients provided written informed consent.

From October 2016 to December 2018, a total of 128 primary knee arthroplasties were performed using the Journey II BCS system. Patients with primary varus OA who underwent knee replacement using the image-free navigation system (Precision N; Stryker Orthopedics, Mahwah, NJ, USA) were included. The exclusion criteria were as follows: presence of a valgus deformity (3 knees); patients in whom a navigation system was not used due to malfunction (4 knees); a diagnosis of osteonecrosis (4 knees); postoperative complications (1 knee: patella clunk syndrome treated with arthroscopic debridement); and complete data not recorded (12 knees).

A total of 104 knees were evaluated retrospectively. Our institution is a highly specialized unit with prospective data collection for all arthroplasty patients. We collected perioperative data of the study population from the hospital’s database retrospectively and performed clinical and radiological evaluation at follow-up. The patient population was composed of 88 women and 16 men (mean age, 73.3 ± 8.3 years; mean body mass index, 27.4 ± 4.2 kg/m^2^; mean preoperative hip–knee–ankle [HKA] angle, 169.1° ± 6.1° [10.9° in varus]).

All procedures were performed by five knee surgeons who used the same surgical technique. A senior surgeon (H.I.) participated in all procedures either as the chief surgeon or first assistant.

### Surgical procedure

A paramedian approach was applied for all patients, and the patella was not everted. The distal femur and proximal tibia were cut guided by the navigation system. Femoral alignment was aimed for placement of 90° to the mechanical axis in the frontal plane and 4° of flexion in the sagittal plane. For the tibia, the alignment was aimed at 90° to the mechanical axis in the frontal plane and 3° of the posterior slope in the sagittal plane.

Soft tissue balancing was achieved, and the extension and flexion gaps were measured using a balancer device. To maintain medial stability, amount of posterior femur resection was adjusted to equalize the extension and flexion gaps in the medial compartment.

Femoral rotation was determined to be parallel to the surgical epicondylar axis, allowing residual lateral ligamentous laxity [9–11]. Tibial rotational alignment was determined using the range of motion (ROM) technique, wherein the knee was put through a full range of flexion and extension, allowing the tibial trial to orientate itself in the best position relative to the femoral component, thereby reducing rotational mismatch of the components [12].

### Intraoperative component gap measurement

After these procedures, the extension and flexion gaps between the osteotomized surfaces were measured twice by the chief surgeon using the same ligament tensioner with a distraction force of 80 N for each compartment and the averages were used. The mean joint gaps at extension and flexion were 22.2 ± 1.8 mm and 22.5 ± 2.0 mm, respectively, in the medial compartment and 24.3 ± 2.3 mm and 24.0 ± 2.4 mm, respectively, in the lateral compartment.

After evaluating soft tissue balance between the osteotomized surfaces, the femoral trial component was placed with the following tensor device on the surface of the tibial bone cut and the patellofemoral joint was reduced. The tensor device consisted of three parts: the upper compartment-specific plates, lower platform plate, and an extra-articular main body. The upper plates had identical shapes of medial and lateral compartments of the polyethylene trial surface of the Journey II BCS system. This device was designed to allow surgeons to measure the joint component gap of the medial and lateral compartments, respectively. Using this tensor device, the component gap of each compartment was assessed at flexion angles of 0°, 30°, 60°, 90°, and 120°, with a joint distraction force of 80 N for each compartment. In our study, the medial and lateral component “laxity” was defined as values of “component gap minus selected thickness of the tibial component” [13].

### Postoperative rehabilitation

The same rehabilitation protocols were applied in all patients. ROM exercise and walking exercise with a crutch and then a walker were started on the first postoperative day. At 2–3 weeks postoperatively, the patient was discharged from our hospital and completed their rehabilitation protocol with physiotherapists.

### Postoperative evaluation

#### Radiographic evaluation

The HKA angle was measured using full-length, standing radiography. Full-length radiography was utilized to measure the frontal alignment of the femoral and tibial components (frontal femoral component [FFC] angle and frontal tibial component [FTC] angle). Lateral radiography was used to measure the femoral component sagittal alignment and tibial slope (lateral femoral component [LFC] angle and lateral tibial component [LTC] angle). Computed tomography images were used to evaluate the rotational alignment of the femoral and tibial components. The rotational femoral component angle was defined as the angle between the line of the anterior cutting surface and the surgical epicondylar axis. The rotational tibial component angle was defined as the angle between the line connecting the medial border of the tibial tuberosity with the center of the posterior concavity of the tibial component and the line passing through the anteroposterior center of the tibial component [14, 15].

To evaluate intraobserver and interobserver reproducibility, the measurements were performed twice by one surgeon (H.I.) at 3-month intervals and once by another examiner (K.K.) who was a knee surgeon with no knowledge of the patients on 50 knees selected randomly from the study group. Intraclass correlation coefficients to measure inter- and intraobserver reliability were high enough for 0.8.

#### Clinical evaluation

Satisfaction score was evaluated using the 2011 Knee Society Scoring (KSS) system [16]　at 1 year postoperatively. The ROM and validated version of the Knee Injury and Osteoarthritis Outcome Score (KOOS) were used to evaluate postoperative clinical scores. The KOOS is a self-reporting questionnaire with 42 items in 5 separately analyzed subscales of pain: symptoms; activities of daily living (ADL); physical, sports, and recreational function; and knee-related quality of life. Each of the five scores was calculated as the sum of the items included, and the scores were then transformed into a 0- to 100-point scale, with 0 points indicating extreme knee problems and 100 points indicating no knee problems [17, 18]. The current study focused on the patient satisfaction score at 1 year postoperatively and did not assessed the long-term clinical results or survivorship.

### Statistical analyses

Data were analyzed using the Bell Curve 2016 (SSRI Co., Ltd., Tokyo, Japan) software package for Microsoft Windows, and tests for normality and distribution were performed using the Kolmogorov–Smirnov test. We compared preoperative and postoperative parameters (ROM, KOOS) using a paired t test. We conducted Pearson’s correlation analysis to assess the correlation between surgical parameters and patient satisfaction and the unpaired t test to compare the patient satisfaction scores between the two groups for qualitative data (female or male, mechanically aligned or not). The relations between the satisfaction score and surgical parameters were calculated using a forced entry multivariate regression analysis. We confirmed the independency of independent variables before including them in the multivariable logistic regression analysis. A power analysis demonstrated that the minimum required number of knee was 83 with estimated R^2^ of 0.15 in order to obtain a power of 0.8 and an alpha of 0.05 for a multivariate regression analysis with 6 explanatory variables. All significance tests were two-tailed, and a significance level of *P* < 0.05 was used for all tests.

## Results

Preoperative and postoperative ROM and KOOS results are presented in Table 1. All parameters improved significantly after the operation. The postoperative satisfaction score according to the 2011 KSS was 28.6 ± 8.1.

**Table 1:** Comparisons of preoperative and postoperative ROM and KOOS scores

	Pre-operation	Post-operation	*P*-value
Maximum flexion (°)	118.4 ± 14.0	123.6 ± 15.6	0.002
Maximum extension (°)	9.1 ± 6.5	0.9 ± 2.3	< 0.001
KOOS			
Pain	47.1 ± 18.3	86.5 ± 13.4	< 0.001
Symptom	54.3 ± 19.1	85.7 ± 11.6	< 0.001
ADL	54.9 ± 15.9	83.9 ± 12.4	< 0.001
Sports	19.6 ± 16.8	52.2 ± 25.9	< 0.001
QOL	25.7 ± 16.2	67.2 ± 22.4	< 0.001

The data are expressed as mean ± standard deviation

*ROM* range of motion; *KOOS* Knee injury and Osteoarthritis Outcome Score; *ADL* activities of daily living; *QOL* quality of life

**Fig. 1:**
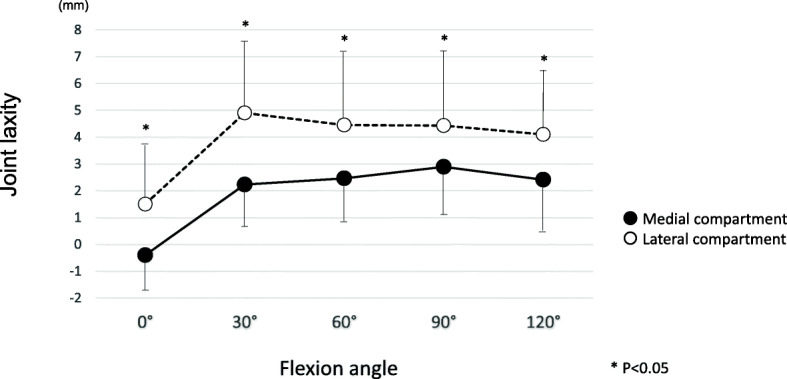
Joint laxity of the medial and lateral compartments at 0°, 30°, 60°, 90° and 120° flexion. Lateral joint laxity is significantly larger than medial joint laxity at each flexion angle (*P* < 0.05)

Figure 1 presents the joint laxity in each compartment. Lateral joint laxity was significantly larger than medial joint laxity at each flexion angle (*P* < 0.05).

The postoperative HKA angle was 179.2° (0.8° in varus) ± 1.9°. The components of 93 patients (89.4 %) were mechanically aligned (within 0° ± 3° of the mechanical axis), whereas those of 11 patients (10.6 %) were outside that range. The mean component alignments were FFC, 89.4° (0.6° in varus) ± 1.8°; FTC, 89.8° (0.2° in varus) ± 1.5°; LFC, 86.4° (3.6° in flexion) ± 2.1°; and LTC, 86.5° (3.5° in posterior slope) ± 1.6°. The rotational femoral component angle was 0.1° ± 1.6° internal rotation, and the rotational tibial component angle was 3.8° ± 4.0° external rotation.

### Univariate analysis for intraoperative factors

**Fig. 2:**
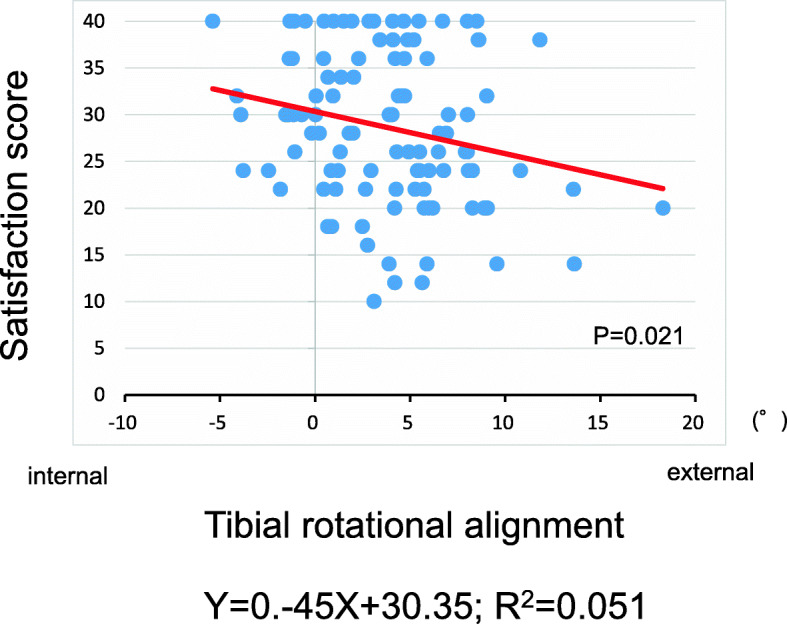
Correlations between satisfaction score of the 2011 Knee Society Scoring System and tibial rotational alignment. Satisfaction score was negatively correlated with tibial external rotation (*R* = 0.22; *P* = 0.021)

**Fig. 3:**
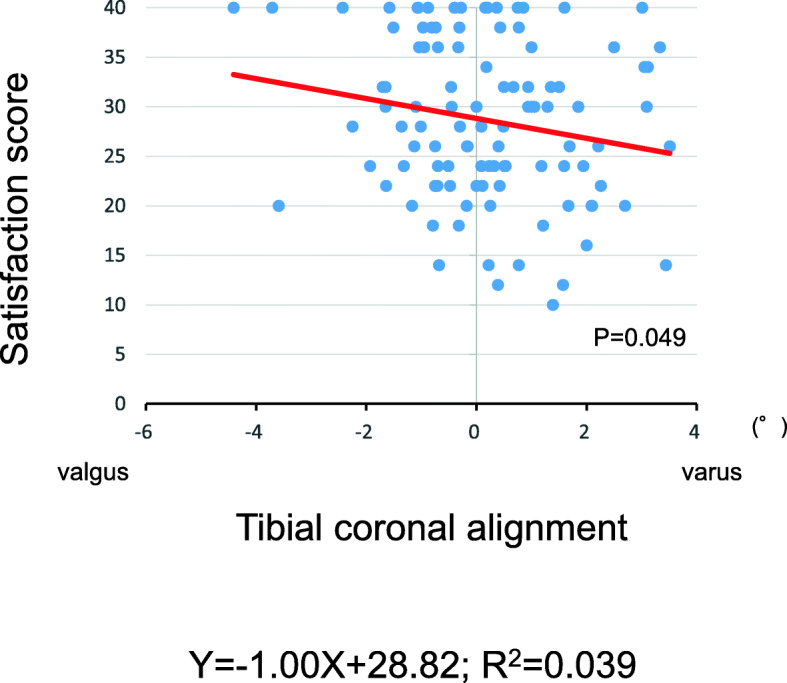
Correlations between satisfaction score of the 2011 Knee Society Scoring System and tibial coronal alignment. Satisfaction score was negatively correlated with tibial varus angle (*R* = 0.20; *P* = 0.049)

**Fig. 4:**
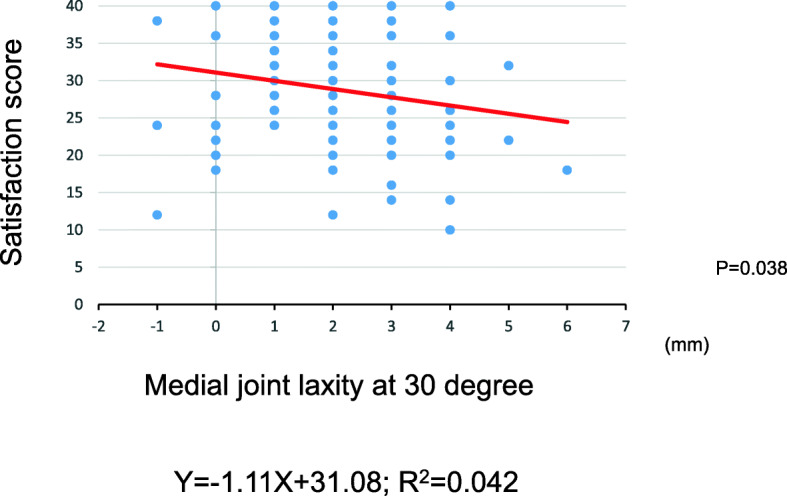
Correlations between satisfaction score of the 2011 Knee Society Scoring System and medial joint laxity at 30° flexion. Satisfaction score was negatively correlated with medial joint laxity at 30° flexion (*R* = 0.21; *P* = 0.038)

**Fig. 5:**
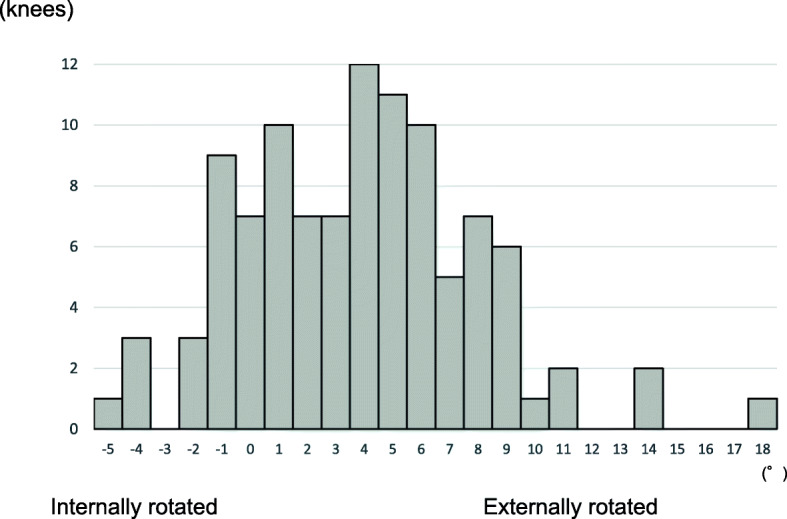
Distribution of tibial rotational angle

**Fig. 6:**
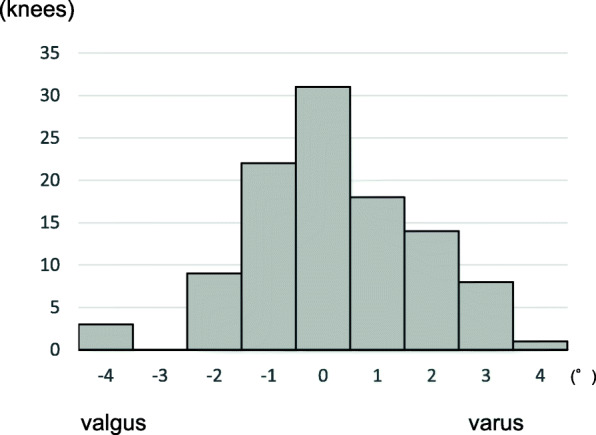
Distribution of tibial varus angle

**Fig. 7:**
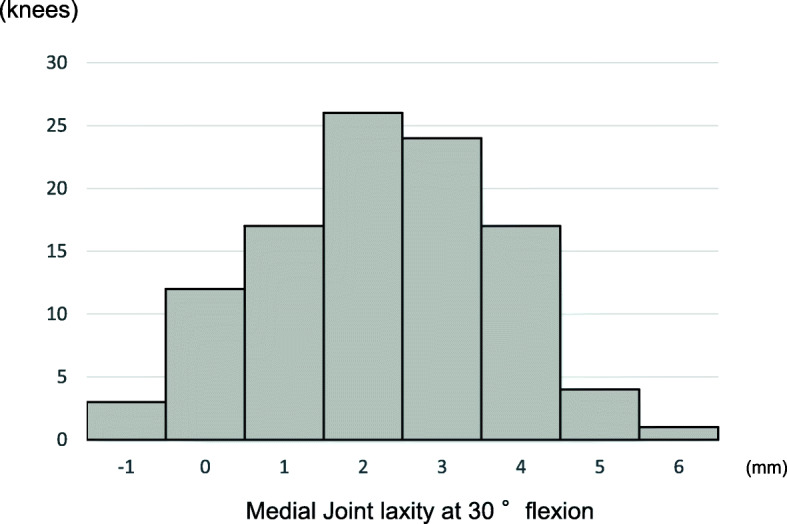
Distribution of the medial joint laxity at 30° flexion

The tibial external rotational angle was negatively correlated with satisfaction (P = 0.021; Fig. 2). The tibial varus angle in the coronal plane was negatively correlated with satisfaction (P = 0.049; Fig. 3). Medial joint laxity at 30° flexion negatively correlated with satisfaction score (P = 0.038; Fig. 4). Figures present the histograms of tibial external rotation angle (Fig. 5), tibial varus angle (Fig. 6) and medial joint laxity at 30° flexion (Fig. 7).

Postoperative HKA, FFC, LFC, LTC, femoral rotational angle, medial joint laxity at 0°, 60°, 90°, 120°, and lateral joint laxity at 0°, 30°, 60°, 90°, and 120° exhibited no significant correlation with satisfaction.

The satisfaction score for men was 31.4 ± 8.7, whereas that for women was 28.1 ± 8.2. There was no significant difference between the two groups (*P* = 0.112). The mean satisfaction score for the mechanically aligned group was 29.0 ± 8.6, whereas that of the outlier group was 25.3 ± 7.7. There was no significant difference between the two groups (*P* = 0.154).

### Multivariate regression analyses

We conducted multivariate regression analyses to evaluate the parameters that affected the satisfaction scores retrospectively. We used age, gender, body mass index, and intraoperative parameters that had correlation with the satisfaction score (*P* < 0.05). Multivariate analysis revealed that medial joint laxity at 30° flexion (*P* = 0.003), tibial excessive external rotational (*P* = 0.009), and tibial varus alignment (*P* = 0.029) were predictors of poor satisfaction score (Table 2, *R*^2^ = 0.19, *P* = 0 0.0019).

**Table 2:** Results of multivariable regression analysis

	Standardized partial regression coefficient	standard error	95 %conficence interval	*P*-value
Age	-0.123	0.095	-0.309–0.688	0.209
Gender (male)	0.127	2.279	-1.673–7.373	0.214
BMI	0.008	0.186	-0.354–0.384	0.936
Medial joint laxity at 30°	-0.209	0.518	-2.631 – -0.572	0.003
Tibial external rotational angle	-0.267	0.198	-0.923 – -0.138	0.009
Tibial varus angle	-0.295	0.498	-2.091 – -0.113	0.029

*BMI* body mass index

## Discussion

The most important finding of this study was that the intraoperative predictors of patient dissatisfaction following BCS TKA were medial joint laxity at 30° flexion, excessive external rotational alignment, and varus alignment of the tibia.

Soft tissue balancing is one of the most important factors for successful TKA [19–21], although studies have reported a poor relationship between the intraoperative soft-tissue balance and clinical outcomes using physician-derived clinical scores [22]. Recently, several articles have reported a relationship between intraoperative soft tissue balance and patient-reported scores. Azukizawa et al. [23] reported that intraoperative medial joint laxity during flexion decreases patient satisfaction following cruciate-sacrificed TKA. The current study also revealed that medial joint laxity in mid-flexion range decreased satisfaction following BCS TKA. Although BCS TKA is reported to alleviate mediolateral instability in flexion range owing to its prosthesis design, excessive laxity in mid-flexion is not appropriate for BCS TKA similar to other prostheses designed for TKA.

Regarding the relationship between the rotational alignment of the tibial component and clinical outcomes, several papers have revealed that internal rotation of the tibial component caused pain and limited motion after TKA [24, 25]. Panni et al. [26] reported that excessive tibial internal rotation, in particular, more than 10° of internal rotation, indicates a significant risk factor for pain and inferior functional outcomes following TKA. However, the current study revealed that internal rotational alignment did not have a risk of poor outcome. This difference might be caused by the fact that there was no case of excessive internal rotation (more than 10°) in the current study.

Contrarily, tibial external rotation has often been reported to have no influence on the outcomes of TKA [26]. However, in the current study excessive external rotation turned out to be a risk factor for poor satisfaction following Journey II BCS TKA. Journey I TKA has been reported to strictly induce internal rotational movement of the tibia relative to femur with flexion by its mechanically constraint-guided motion system [27]. Therefore, when the tibial component is implanted in an excessively externally rotated manner, excessive internal rotational movement of the tibia relative to femur will be induced with flexion and lead to severe loading to the ITB [28]. The modification of the Journey I BCS to Journey II BCS system has reduced the complications including ITB syndrome and dislocations by making the amount of femorotibial rotational angle and anterior-posterior translation smaller than normal knees during flexion, while maintaining the normal-like movements such as medial pivot motion and bicondylar roll back movement [29]. However, even when using modified Journey II BCS prosthesis, excessive external rotation (more than 10°) of the tibia component might induce the excessive traction force to the ITB during flexion and reduce the satisfaction score.

Accurate limb alignment is also essential for a successful TKA. It is one of the most commonly accepted principles of TKA that one should aim for mechanical alignment [30, 31]. Recently, the importance of mechanical alignment has been questioned [32–34], and several studies have shown that some mild varus alignment after TKA leads to better results for patients with varus OA [35, 36]. In the current study, we included 11 patients with varus alignment (more than 3°), whose average satisfaction score was 25.3 ± 7.7, whereas the satisfaction score of the mechanically aligned group was 29.0 ± 8.6. Furthermore, tibial varus alignment leads to significantly inferior satisfaction scores. The BCS prosthesis replicates 3° of the tibial varus angle with an asymmetrical tibial plateau and a medially concave and laterally convex shape relative to the tibial baseplate using 2.5-mm differences in the medial and lateral compartment thickness. Therefore, when the distal femur and proximal tibia are cut perpendicular to the mechanical axis in the frontal plane, the component alignment will be “anatomical alignment” [37]. Anatomical alignment techniques produced good clinical results [38, 39]. Regarding BCS TKA, additional alignment adjustment is not necessary, or rather, it reduces clinical outcomes.

The mean 2011 KSS score indicating satisfaction following BCS TKA was 28.6 ± 8.1. Patient satisfaction following TKA is reported to differ by race [40]. Several reports from Asia assessed patient satisfaction using the 2011 KSS. The reported satisfaction scores of other TKA designs (posterior stabilized, cruciate retaining, and cruciate sacrificing) ranged from 21 to 26 [35, 41–45]. To know whether BCS TKA is truly superior to other TKA designs, we must compare the satisfaction scores between BCS and other designs of TKAs in patients with similar demographics.

There are several limitations. First, this study was retrospective. Second, the follow-up period was relatively short. We have never experienced a problem with loosening, polyethylene wear or breakage of either TKA designs. However, BCS TKA behaves strictly as a mechanically constraint-guided motion system. A lot of stress on the polyethylene insert might cause the future problems. Third, we investigated only varus OA knees in the current study. Therefore, our results cannot be applied to valgus knees. Further research using valgus knees should be done, with the additional factor of whether or not the valgus knee is included in the surgical indication of BCS TKA. Fourth, we did not assess preoperative KOOS scores. Preoperative KOOS scores might be important and necessary. However preoperative KOOS questionary was not carried out for all the patients. Therefore, we couldn’t use preoperative KOOS as a parameter in this multivariate regression analysis. Fifth we did not evaluate mental health which has been reported to affect the patient satisfaction after TKA. Patient satisfaction is multifactorial with some factors beyond the scope of a surgeon’s control including preoperative patient reported function, narcotic use, mental health, expectation and lumber spine pain [46–48]. Further studies are needed to identify the preoperative and postoperative predictors of patient satisfaction following BCS TKA.

## Conclusions

In conclusion, the current retrospective multiple regression analysis revealed that medial joint laxity at 30° flexion, tibial excessive external rotational, and tibial varus alignment were predictors of poor satisfaction score after BCS TKA.

## Data Availability

The datasets used during the current study are available from the corresponding author on reasonable request.

## Abbreviations

TKA: Total knee arthroplasty
BCS: Bi-cruciate stabilized
OA: Osteoarthritis
ITB: Iliotibial band
HKA: Hip knee ankle
ROM: Range of motion
FFC: Frontal femoral component
FTC: Frontal tibial component
LFC: Lateral femoral component
LTC: Lateral tibial component
KSS: Knee society scoring
KOOS: Knee injury and osteoarthritis outcome score
ADL: Activities of daily living
